# Cultivar differences in heat tolerance of *Oncidium* orchids: physiological mechanisms and implications for breeding strategies

**DOI:** 10.3389/fpls.2026.1831843

**Published:** 2026-05-22

**Authors:** Xiaoyan Luo, Xiaoyu Hong, Yuanhua Luo, Shuangshuang Yi, Xiaoyun Yu, Chonghui Li, Yi Liao, Mengjin Zhang, Shunjiao Lu

**Affiliations:** 1 Tropical Crops Genetic Resources Institute, Chinese Academy of Tropical Agricultural Sciences, Haikou, China; 2 Key Laboratory of Crop Gene Resources and Germplasm Enhancement in Southern China, Ministry of Agriculture, Haikou, China; 3 Engineering Technology Research Center of Tropical Ornamental Plant Germplasm Innovation and Utilization, Haikou, China; 4 Key Laboratory of Tropical Crops Germplasm Resources Genetic Improvement and Innovation of Hainan Province, Haikou, China; 5 Fujian Academy of Agricultural Sciences, Crop Research Institute, Fuzhou, Fujian, China; 6 Sanya Academy of Forestry, Sanya, China

**Keywords:** antioxidant enzymes, heat tolerance, high temperature stress, membrane stability, *Oncidium* orchids, osmotic adjustment, physiological biomarkers

## Abstract

**Background:**

*Oncidium* orchids are a core economic genus in the global tropical ornamental flower industry, while sustained high temperature has become the key bottleneck restricting its commercial production. The coordinated physiological mechanism of heat adaptation, unified screening temperature threshold, and practical physiological biomarkers for heat tolerance evaluation in *Oncidium* remain insufficiently characterized.

**Methods:**

This study systematically evaluated the heat tolerance of 36 *Oncidium* cultivars through natural field heatwave screening, controlled environment validation, and physiological mechanism dissection under graded temperature stress.

**Results:**

We screened 8 highly heat-tolerant cultivars from 36 tested materials, and identified 40.00°C as the optimal discriminative temperature for heat tolerance screening. Relative electrolyte conductivity (REC), proline content, and peroxidase (POD) activity were confirmed as the core physiological biomarkers (coefficient of variation > 50.00%). Principal component analysis revealed that membrane stability and osmotic adjustment were the primary determinants of *Oncidium* heat adaptation, explaining 80.95% of the total variance. Notably, our data suggested that excessive proline accumulation in heat-sensitive cultivars was a passive compensatory response after cell damage, rather than an active protective adaptation, which differs from the conventional view that higher proline accumulation indicates stronger stress tolerance.

**Conclusion:**

These findings characterize physiological responses associated with heat tolerance in *Oncidium* and provide practical reference markers and a physiological basis for future genetic and molecular studies in orchids.

## 1 Introduction

**Figure 1:**
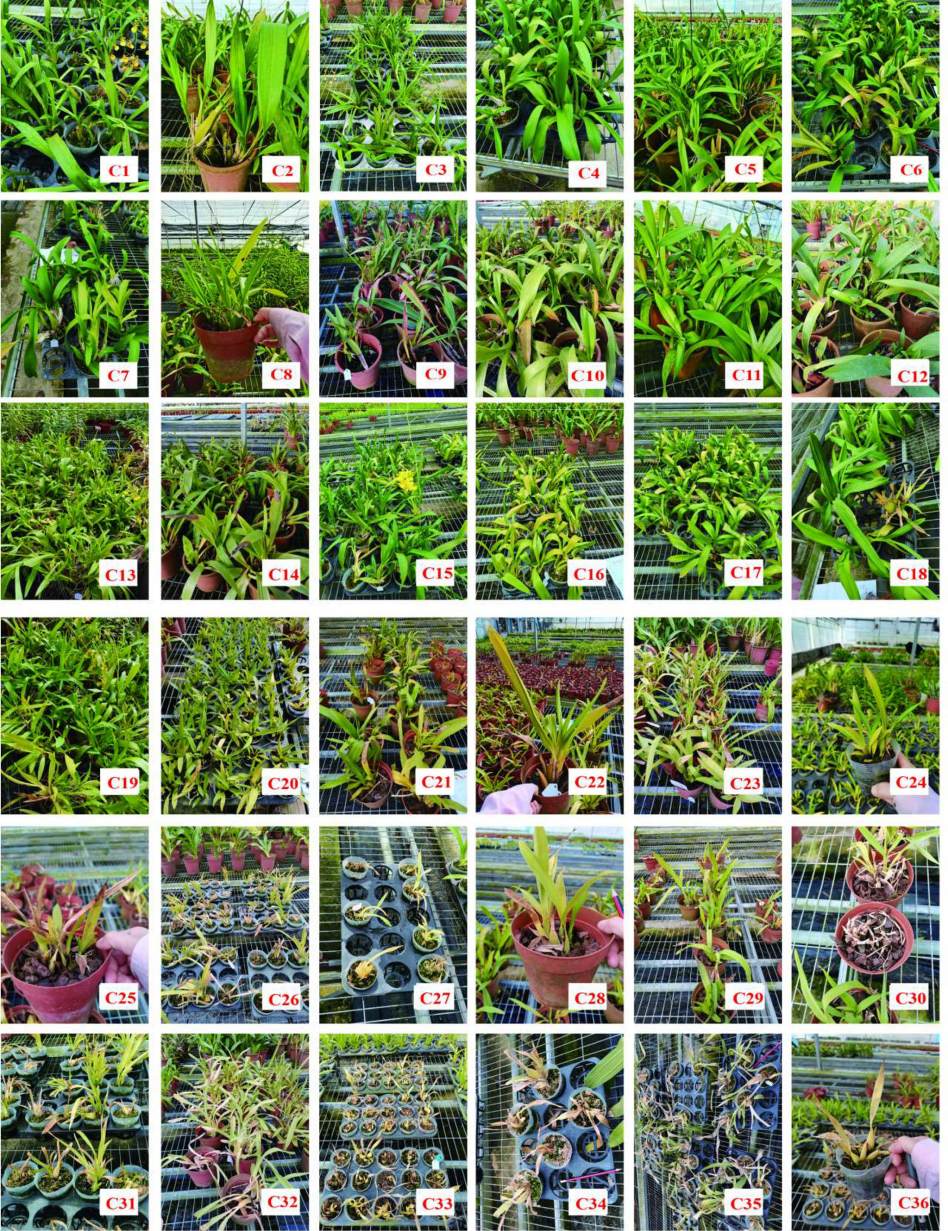
Field heat damage performance of *Oncidium* in the *Oncidium* germplasm conservation nursery in Danzhou, Hainan Province under continuous high temperature stress in the summer of 2021.

*Oncidium* orchids are a core economic genus in the global tropical ornamental flower industry. Global climate change and frequent extreme heatwaves have severely restricted the sustainable development of the horticultural industry worldwide (Bita and Gerats, 2013; Chadha, 2015). In major production regions such as Hainan, China, sustained temperatures above 35.00 °C cause severe growth inhibition, flower spike degradation, and commercial trait deterioration in *Oncidium*, directly leading to 30.00–50.00% loss of marketable products, which has become the key bottleneck restricting the quality and efficiency improvement of the tropical orchid industry (Hew and Yong, 2004). This vulnerability was dramatically validated during the 2021 heatwave event at the Tropical Flower Research Center of the Chinese Academy of Tropical Agricultural Sciences (Danzhou, Hainan Province): approximately 2,000 seedlings from more than 60 *Oncidium* cultivars suffered severe heat damage, with significant phenotypic differences among varieties: heat-tolerant cultivars maintained vigorous growth, while heat-sensitive ones exhibited severe leaf chlorosis, wilting, and even complete mortality (**Figure 1**). These field observations indicate the need for systematic research on the heat tolerance mechanism and standardized evaluation method for *Oncidium*.

Recent studies have significantly advanced the understanding of physiological mechanisms underlying orchid heat tolerance. In *Dendrobium nobile*, heat stress triggers reactive oxygen species (ROS) accumulation, which is mitigated by enhanced activities of antioxidant enzymes including superoxide dismutase (SOD) and peroxidase (POD), as well as the synthesis of osmoprotectants such as proline and soluble sugars; exogenous calcium has also been confirmed to improve its heat tolerance by stabilizing membrane integrity (Fan et al., 2023). Similarly, studies on *Phalaenopsis* have identified genetic loci and key physiological traits (e.g., sustained photosynthetic efficiency) associated with heat tolerance, providing a reference for heat tolerance research in *Orchidaceae* (Blischak, 2005), and related biochemical and molecular regulatory mechanisms have also been verified in other orchid genera such as *Cymbidium* (Balilashaki et al., 2022).

In contrast, studies specifically focusing on *Oncidiu*m are limited and fragmented. Our previous study preliminarily screened the phenotypic heat tolerance of 38 *Oncidium* cultivars under natural high temperature stress, and constructed a preliminary phenotypic evaluation system for heat tolerance (Luo et al., 2024). However, existing limited reports (including our previous work) confirmed only the differences in membrane stability (measured as relative electrolyte conductivity, REC) and proline accumulation among partial *Oncidium* cultivars under high temperature (Sembi et al., 2019; Masouleh and Sassine, 2020; Luo et al., 2024), key knowledge gaps remain in this research field. First, an integrated understanding of multi-physiological traits associated with *Oncidium* heat tolerance is lacking; existing studies on individual indicators cannot clarify the contribution of different physiological pathways to heat adaptation, limiting the in-depth understanding of the heat response mechanism in *Oncidium*. Second, no unified temperature threshold and treatment protocol for heat tolerance identification of *Oncidium* have been established; inconsistent stress conditions in existing studies make it difficult to horizontally compare the heat tolerance of different cultivars, limiting the efficient screening and utilization of germplasm resources. Third, practical physiological biomarkers suitable for rapid heat tolerance screening are still lacking, which is not conducive to the efficient identification of heat tolerance in breeding populations of *Oncidium*.

To address these gaps, this study aimed to: (1) evaluate the heat tolerance of 36 *Oncidium* cultivars under natural field heatwave and controlled high-temperature conditions; (2) examine physiological responses—including water relations, membrane stability, osmotic adjustment, and antioxidant defense—associated with differential heat tolerance among cultivars; (3) identify the temperature threshold and physiological markers that can be used to assess heat tolerance in *Oncidium*.

## 2 Materials and methods

### 2.1 Preliminary evaluation of heat tolerance in 36 *Oncidium* cultivars under field conditions

This study was conducted at the *Oncidium* germplasm conservation nursery in the Tropical Flower Research Center, Chinese Academy of Tropical Agricultural Sciences (CATAS), Danzhou, Hainan Province, China (19°31’N, 109°34’E). The nursery consisted of standard rain-shelter film greenhouses with a closed structure covered by rainproof polyethylene film, where natural ventilation was supplemented by a fan and wet curtain cooling system. A uniform cultivation substrate consisting of coconut shell (particle size 1–2 cm) and sphagnum moss at a volume ratio of 3:1 was used for all plant materials. The irrigation system adopted spray irrigation, with tap water applied once every 2–3 days and adjusted according to real-time weather conditions to maintain the substrate moisture content at 60–70%.

All 36 *Oncidium* cultivars evaluated in this study were core germplasm resources conserved in the National Tropical Orchid Germplasm Resource Bank of the center. These cultivars were introduced from multiple provinces in China, including Fujian, Guangdong, and Yunnan, and had been maintained under the above uniform field management in the nursery for more than 6 months prior to the high-temperature event. Detailed information on the 36 cultivars, including germplasm source, propagation method, seedling age, cultivation substrate, and growth conditions before heat stress, is provided in **Supplementary Table 1**.

Following the prolonged natural high-temperature event in the summer of 2021 (Wu et al., 2022), severe heat damage was observed across all *Oncidium* plants in the nursery, with extremely significant phenotypic differences in heat injury detected among different *Oncidium* cultivars. Taking this natural high-temperature stress event as an opportunity, we performed an *in-situ* preliminary evaluation of heat tolerance on these 36 commonly cultivated *Oncidium* cultivars, by assessing their growth status after the heat damage event.The specific evaluation method and classification criteria were as follows: after the prolonged heatwave, the survival rate of each cultivar was counted and calculated.

Surviving plants were defined as those with fresh heart leaves, no whole-plant wilting or necrosis, and the ability to resume growth after the heatwave; plants with whole-plant browning necrosis and heart leaf necrosis were defined as dead. The survival rate was calculated using the following formula: survival rate = number of surviving plants/total number of plants × 100.00%. Based on the survival rate, all cultivars were classified into five heat tolerance categories: highly heat-tolerant (survival rate ≥ 95.00%), heat-tolerant (80.00–94.00%), moderately heat-tolerant (60.00–79.00%), heat-sensitive (30.00–59.00%), and highly heat-sensitive (survival rate < 30.00%).

Detailed information on the germplasm conservation environment, natural high-temperature stress event background, and preliminary phenotypic observation of the tested materials has been reported in our previous study (Luo et al., 2024). On this basis, this study reclassified the heat tolerance grade of 36 core commercial *Oncidium* cultivars using uniform survival rate criteria, to screen representative materials for subsequent controlled environment validation and in-depth physiological mechanism dissection.

### 2.2 Controlled heat stress experiments and phenotypic validation of nine selected cultivars

Due to the inconsistent seedling ages, diverse introduction origins, variable pre-growth status of the germplasms preserved in the nursery, non-uniform cultivation substrates, and the small sample sizes of some cultivars caused by germplasm resource limitations, the survival rates recorded after the natural heatwave in Section 2.1 were affected by multiple confounding factors and thus could not objectively reflect the inherent heat tolerance of the cultivars themselves. Therefore, under the premise of uniform seedling ages, growth environments, and cultivation substrate, a 28-day continuous high-temperature stress experiment (42.00°C/38.00°C, day/night) was conducted on nine representative cultivars that exhibited contrasting heat tolerance performance in the preliminary field investigation. This experiment was designed to achieve two objectives: (1) to validate the previous heat tolerance evaluation results, and (2) to screen suitable materials for subsequent research on physiological responses to heat stress.

The selection criteria were as follows: all selected cultivars were the main commercial varieties with large-scale promotion in the tropical orchid market, with clear genetic background and typical field heat stress phenotypes. Specifically, the final panel included 2 cultivars from the highly heat-tolerant grade (C5, C11), 3 cultivars from the heat-tolerant grade (C4, C12 and C15), 1 cultivar from the heat-sensitive grade (C32), and 3 cultivars from the highly heat-sensitive grade (C27, C30 and C35).

The plant materials used in this experiment were a new batch of uniform tissue culture seedlings provided by Hainan *Oncidium* Biotechnology Co., Ltd. All seedlings were 8–9 months old with consistent growth status (4–6 fully expanded leaves, no pseudobulb damage or disease), and cultivated in a standard glass greenhouse before the experiment. During the pre-cultivation period, the greenhouse temperature was stably controlled at 22.00 - 28.00°C, and the seedlings were never exposed to high temperature stress or other abiotic stresses at the seedling stage, to ensure the consistency of the initial physiological state of all test materials.

Plants were acclimated in intelligent growth chambers (Percival Scientific, USA) at 22.00°C/25.00°C (day/night) for 7 days before stress treatment to eliminate environmental differences. The acclimation conditions were consistent with the pre-cultivation environment: 240 μmol m^-^² s^-^¹ photosynthetic photon flux density, 14/10 h (day/night) photoperiod, and 80.00% relative humidity. After acclimation, plants were subjected to a 28-day continuous high-temperature stress treatment at 42.00°C/38.00°C (day/night), with other growth conditions completely consistent with the acclimation period.

Phenotypic responses were assessed weekly after the onset of stress by measuring the leaf yellowing rate (YR) and defoliation rate (DR). Yellow leaves were defined as leaves with chlorosis and yellowing area accounting for ≥ 50% of the total leaf area; defoliation rate was calculated based on the number of naturally abscised leaves from the plant during the stress period. The calculation formulas were as follows:YR = number of yellow leaves/total number of leaves × 100.00%;DR = number offallen leaves/total number of leaves × 100.00%. Three biological replicates were set for each cultivar, with 10 plants per biological replicate.

### 2.3 Physiological measurements under graded temperature stress

To elucidate the physiological mechanisms underlying the differential heat tolerance of *Oncidium*, four representative cultivars with distinct heat tolerance gradients were selected from the nine cultivars used in the controlled validation experiment in Section 2.2: C11 (highly heat-tolerant), C4 (heat-tolerant), C32 (heat-sensitive), and C27 (highly heat-sensitive). The selection criteria were as follows: one representative cultivar was selected from each of the four heat tolerance grades, which exhibited the most typical phenotypic performance in the controlled environment experiment. The four selected cultivars represented the full range of heat tolerance among the 36 tested cultivars: C11 (100.00% survival, highly heat-tolerant), C4 (93.04%, heat-tolerant), C32 (41.67%, heat-sensitive), and C27 (14.28%, highly heat-sensitive). Pairwise comparisons of YR and DR confirmed their statistically distinct heat tolerance phenotypes (**Table 1**; **Supplementary Table 5**).The plant materials used in this experiment were from the same batch of uniform tissue culture seedlings as those in Section 2.2, which were purchased from Hainan *Oncidium* Biotechnology Co., Ltd.

**Table 1:** The leaf yellowing and fallen rate of 9 *Oncidium* cultivars under high temperature stress.

Cultivar	Rate type	0 week	1 week	2 weeks	3 weeks	4 weeks
C4	DR	0.00 ^± 0.00^	0.00 ^± 0.00^	0.00 ^± 0.00^	0.00 ^± 0.00^	0.00 ^± 0.00^
	YR	0.00 ^± 0.00^	0.00 ^± 0.00^	4.80 ^± 0.80^	61.40 ^± 5.90^	74.50 ^± 2.50^
C5	DR	0.00 ^± 0.00^	0.00 ^± 0.00^	2.80 ^± 0.80^	5.60 ^± 0.60^	5.60 ^± 1.60^
	YR	0.00 ^± 0.00^	0.00 ^± 0.00^	2.80 ^± 0.80^	7.90 ^± 0.80^	10.70 ^± 0.40^
C11	DR	0.00 ^± 0.00^	0.00 ^± 0.00^	0.00 ^± 0.00^	0.00 ^± 0.00^	0.00 ^± 0.00^
	YR	0.00 ^± 0.00^	0.00 ^± 0.00^	2.40 ^± 0.40^	2.40 ^± 0.40^	2.40 ^± 0.40^
C12	DR	0.00 ^± 0.00^	0.00 ^± 0.00^	0.00 ^± 0.00^	0.00 ^± 0.00^	0.00 ^± 0.00^
	YR	0.00 ^± 0.00^	0.00 ^± 0.00^	3.30 ^± 0.30^	17.00 ^± 8.20^	84.90 ^± 8.70^
C15	DR	0.00 ^± 0.00^	0.00 ^± 0.00^	39.30 ^± 2.70^	80.01 ^± 0.20^	88.90 ^± 11.00^
	YR	0.00 ^± 0.00^	0.00 ^± 0.00^	63.50 ^± 1.10^	100.00 ^± 0.00^	100.00 ^± 0.00^
C27	DR	0.00 ^± 0.00^	0.00 ^± 0.00^	0.00 ^± 0.00^	2.00 ^± 0.20^	2.00 ^± 0.20^
	YR	0.00 ^± 0.00^	4.30 ^± 0.20^	91.00 ^± 4.60^	100.00 ^± 0.00^	100.00 ^± 0.00^
C30	DR	0.00 ^± 0.00^	0.00 ^± 0.00^	2.20 ^± 0.20^	2.20 ^± 0.20^	6.70 ^± 1.70^
	YR	0.00 ^± 0.00^	0.00 ^± 0.00^	70.50 ^± 8.70^	100.00 ^± 0.0^	100.00 ^± 0.0^
C32	DR	0.00 ^± 0.00^	0.00 ^± 0.00^	0.00 ^± 0.00^	11.90 ^± 1.90^	21.40 ^± 5.40^
	YR	0.00 ^± 0.00^	0.00 ^± 0.00^	65.40 ^± 7.30^	100.00 ^± 0.0^	100.00 ^± 0.0^
C35	DR	0.00 ^± 0.00^	0.00 ^± 0.00^	0.00 ^± 0.00^	13.30 ^± 0.30^	13.00 ^± 0.30^
	YR	0.00 ^± 0.00^	0.00 ^± 0.00^	100.00 ^± 0.00^	100.00 ^± 0.00^	100.00 ^± 0.00^

YR, Leaf Yellowing Rate; DR, Defoliation Rate. % ± SD of 9 *Oncidium* cultivars under continuous high-temperature stress (42.00 °C/38.00 °C day/night). Data were recorded weekly over four weeks (n=3 biological replicates per time point).

Plants were first acclimated in intelligent growth chambers (Percival Scientific, USA) at 22.00°C for 7 days, with acclimation conditions consistent with the pre-cultivation environment: 240 μmol m^-^² s^-^¹ photosynthetic photon flux density, 14/10 h (day/night) photoperiod, and 80.00% relative humidity. After acclimation, plants were exposed to three graded high-temperature stress treatments: moderate (35.00°C), critical (40.00°C), and extreme (45.00°C). For each temperature treatment, the data collected at 0 hour (immediately before the onset of stress) was used as the control. Leaf samples were collected from the 1st to 2nd newly expanded young leaves on the newly formed pseudobulbs of plants with consistent growth, avoiding leaf veins and sites with pest infestation or disease damage; 3 independent biological replicates were set for each temperature treatment and each sampling time point, with every 3 plants pooled as one biological replicate. Leaf samples were collected at 0, 2, 4, 8, 12, 24, and 48 hours after the onset of each temperature treatment. However, due to severe desiccation and tissue necrosis of plants under the extreme 45.00 °C treatment, sampling at the 48-hour time point was omitted for this temperature gradient.

The detailed measurement methods for each physiological index are described as follows: relative electrolyte conductivity (REC) was measured according to the method described by (Seitz et al., 2019); leaf water content (LWC) was determined by gravimetric method, where the fresh weight (FW) of leaf samples was weighed immediately after sampling, the samples were fixed at 105 °C for 30 min and then dried at 80 °C to constant weight to obtain the dry weight (DW), and LWC was calculated using the formula: LWC (%) = (FW - DW)/FW × 100%; malondialdehyde (MDA) content was determined using the thiobarbituric acid method with BC0025 kit, proline content was determined using the acid ninhydrin method with BC0290 kit (both kits were purchased from Solarbio Life Sciences Co., Ltd., Beijing, China), and soluble sugar content was determined by anthrone-sulfuric acid method, with all operations carried out in strict accordance with the kit instructions and standard experimental protocols; for antioxidant enzyme activity, peroxidase (POD) activity was assayed by guaiacol oxidation method at 470 nm, with 1 U defined as the amount of enzyme causing a 0.01 increase in absorbance per minute, while catalase (CAT) activity was assayed by H_2_O_2_ decomposition method at 240 nm, with 1 U defined as the amount of enzyme decomposing 1 μmol of H_2_O_2_ per minute, and all indexes were assayed with three technical replicates for each biological replicate.

### 2.4 Data analysis

Data were analyzed using SPSS 26.0 (IBM Corp., Armonk, NY, USA) and R 4.2.1 (R Core Team, Vienna, Austria). Missing data for the 45.00 °C/48 h time point (due to severe plant desiccation and tissue necrosis) were excluded from the analysis prior to formal data processing. Prior to statistical analysis, the Shapiro–Wilk test was used to verify the normality of data, and Levene’s test was used to verify the homogeneity of variance. Data conforming to normal distribution and homogeneity of variance were used for subsequent parametric analysis; data that did not meet the above assumptions were subjected to logarithmic transformation for normalization, or analyzed using the non-parametric Kruskal-Wallis test followed by Dunn’s *post-hoc* test for multiple comparisons. The effects of three fixed factors—cultivar (genotype), temperature (stress intensity), and treatment time (stress duration)—on physiological parameters were assessed by three-way analysis of variance (ANOVA) with Type III sums of squares. Leaf age (node position) was included as a covariate in the three-way ANOVA to account for potential developmental heterogeneity. The updated ANOVA results are presented in **Supplementary Table 4**. Mean separation was conducted using *post-hoc* Tukey’s HSD test at p < 0.05.

Pearson correlation analysis was performed to examine the relationships among physiological traits. Coefficient of variation (CV) analysis was used to identify key discriminative parameters and the optimal screening temperature. Principal component analysis (PCA) was performed after Z-score standardization of all physiological indicators to eliminate the influence of different dimensions; principal components with eigenvalue > 1.00 were extracted according to the Kaiser criterion. R software packages used for data analysis included ggplot2 (for graphing), vegan (for PCA), corrplot (for correlation matrix visualization), and car (for three-way ANOVA).

## 3 Results

### 3.1 Evaluation of heat tolerance in *Oncidium* cultivars under natural high-temperature conditions

Through natural heatwave stress, 8 *Oncidium* cultivars with a field survival rate ≥ 95.00% were identified from 36 germplasms. These cultivars may be useful as parental materials in breeding programs. The field trial was conducted in Danzhou, Hainan Province, where the maximum daily temperature reached 36.00–37.00 °C in the summer of 2021, with an extreme peak of 41.40 °C (Wu et al., 2022), and the heat tolerance of all cultivars was systematically evaluated based on survival rate.

Consistent with the phenotypic variation shown in **Figure 1** (Introduction), the results revealed extremely significant genotypic differences in heat tolerance among the 36 *Oncidium* cultivars (**Table 2**). Based on survival rates, the 36 cultivars were classified into five distinct heat tolerance categories: highly heat–tolerant (survival rate ≥ 95.00%), heat–tolerant (80.00–94.00%), moderately heat–tolerant (60.00–79.00%), heat–sensitive (30.00–59.00%), and highly heat–sensitive (survival rate < 30.00%). Accordingly, the screening identified 8 highly heat–tolerant cultivars, 7 heat–tolerant cultivars, 5 moderately heat–tolerant cultivars, 9 heat–sensitive cultivars, and 7 highly heat–sensitive cultivars (**Table 2**).

**Table 2:** Classification of 36 *Oncidium* cultivars based on the survival rate under natural high-temperature conditions in Danzhou, Hainan Province.

Cultivar No.	Total plants	Survived	Survival rate (%)	Heat tolerance level
C1	15	15	100.00%	Highly Heat–Tolerant
C2	11	11	100.00%	Highly Heat–Tolerant
C3	23	23	100.00%	Highly Heat–Tolerant
C4	115	107	93.04%	Heat–Tolerant
C5	26	25	96.15%	Highly Heat–Tolerant
C6	37	36	97.30%	Highly Heat–Tolerant
C7	7	7	100.00%	Highly Heat–Tolerant
C8	63	62	98.41%	Highly Heat–Tolerant
C9	36	34	94.44%	Heat–Tolerant
C10	44	41	93.18%	Heat–Tolerant
C11	26	26	100.00%	Highly Heat–Tolerant
C12	13	12	92.31%	Heat–Tolerant
C13	34	29	85.29%	Heat–Tolerant
C14	16	13	81.25%	Heat–Tolerant
C15	42	35	83.33%	Heat–Tolerant
C16	26	21	80.77%	Heat–tolerant
C17	24	19	79.17%	Moderately Heat–Tolerant
C18	14	11	75.00%	Moderately Heat–Tolerant
C19	117	84	71.43%	Moderately Heat–Tolerant
C20	168	115	68.45%	Moderately Heat–Tolerant
C21	14	8	57.14%	Heat–Sensitive
C22	34	16	45.83%	Heat–Sensitive
C23	24	11	45.83%	Heat–Sensitive
C24	119	53	44.44%	Heat–Sensitive
C25	16	7	43.75%	Heat–Sensitive
C26	37	16	43.24%	Heat–Sensitive
C27	7	1	14.28%	Highly Heat–Sensitive
C28	56	22	39.29%	Heat–Sensitive
C29	49	16	32.65%	Heat–Sensitive
C30	18	5	27.78%	Highly Heat–Sensitive
C31	22	5	22.71%	Highly Heat–Sensitive
C32	24	10	41.67%	Heat–Sensitive
C33	24	0	0.00%	Highly Heat–Sensitive
C34	38	0	0.00%	Highly Heat–Sensitive
C35	6	0	0.00%	Highly Heat–Sensitive
C36	16	0	0.00%	Highly Heat–Sensitive

Cultivars were classified into five categories: highly heat–tolerant (survival rate ≥ 95.00%), heat–tolerant (80.00–94.00%), moderately heat–tolerant (60.00–79.00%), heat–sensitive (30.00–59.00%), and highly heat–sensitive (survival rate < 30.00%). The screening identified 8 highly heat–tolerant cultivars, 7 heat–tolerant cultivars, 5 moderately heat–tolerant cultivars, 9 heat–sensitive cultivars, and 7 highly heat–sensitive cultivars. Some cultivars have a small sample size due to the limitation of the existing germplasm resource reserve, and the heat tolerance will be further verified by expanding the sample size in the future.

### 3.2 Validation of heat tolerance in nine selected *Oncidium* cultivars under controlled conditions

Owing to the inconsistent seedling ages, diverse introduction origins, variable pre-growth status of germplasms preserved in the nursery, as well as the non-uniform cultivation substrates (including sphagnum moss and pine bark) of the materials, and the small sample sizes of some cultivars due to germplasm resource reserve limitations, the survival rate after the heatwave was affected by multiple confounding factors and thus could not objectively reflect the inherent heat tolerance of the cultivars themselves. To further evaluate the robustness of the field classification despite these limitations, we performed a sensitivity analysis by excluding six cultivars with sample size < 10 (C7, C14, C19, C28, C33, C35). The remaining 30 cultivars showed 100% consistent classification with the original results (**Supplementary Table 2**). Additionally, 95% confidence intervals for survival rates were calculated using 1000 bootstrap resampling replicates, confirming that the 95% confidence intervals of survival rates between adjacent heat tolerance classes did not overlap (**Supplementary Table 1**).

Therefore, under the premise of uniform seedling ages, growth environments and a consistent cultivation substrate, a 28-day continuous high-temperature stress experiment (42.00 °C/38.00 °C, day/night) was conducted on nine selected representative cultivars that exhibited significantly contrasting heat tolerance performance in the preliminary field investigation under controlled high-temperature stress conditions.

**Figure 2:**
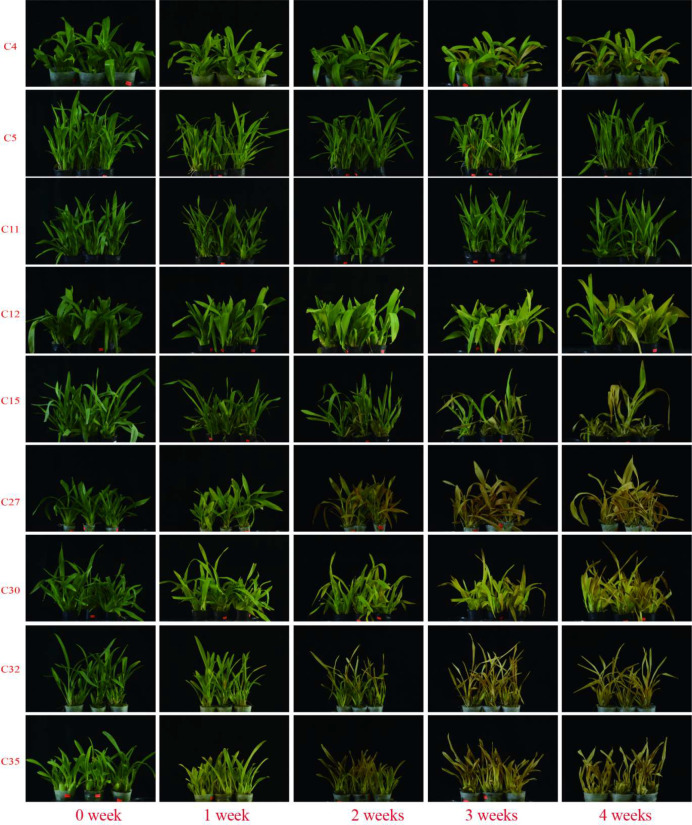
Phenotypes of 9 *Oncidium* cultivars under high-temperature stress.

The results showed that the phenotypic observations of the nine cultivars under sustained extreme high temperature were generally consistent with the initial field classification (**Figure 2**), indicating that the field screening results have referential value for breeding applications. The highly heat-tolerant cultivars (C5 and C11) maintained bright green foliage and upright growth with almost no visible damage throughout the four-week stress period. In contrast, the highly heat-sensitive cultivars (C27, C30, C35) and heat-sensitive cultivar C32 developed severe leaf yellowing within two weeks, with complete leaf desiccation and scorching observed by the fourth week. The highly heat-sensitive cultivar C30 exhibited rapid symptom development, with a leaf yellowing rate of 70.50% at two weeks and 100.00% at three weeks. The heat-tolerant cultivars C4, C12 and C15 (classified as heat-tolerant in the field trial) showed a delayed onset of visible symptoms under controlled stress, yet the severity of their responses varied remarkably.

Notably, C15 exhibited rapid and severe leaf yellowing (63.50%) at two weeks and a high leaf defoliation rate of 88.90% by the fourth week, which was in sharp contrast to its heat-tolerant classification in the field. This indicated that the heat tolerance of C15 was more dependent on diurnal temperature fluctuations in the field rather than sustained extreme high temperature.

Quantitative analysis of the leaf yellowing rate (YR) and defoliation rate (DR) confirmed these phenotypic trends (**Table 1**). After four weeks of stress, the highly heat-tolerant cultivars C5 and C11 had the lowest YR (≤10.70%) and DR (≤5.60%), exhibiting the most stable growth under sustained high temperature. The heat-tolerant cultivars C4 and C12 showed intermediate final YR values (74.50% and 84.90%, respectively) with negligible DR (0.00% for both), maintaining good plant integrity despite leaf yellowing. In stark contrast, five cultivars (heat-tolerant C15, highly heat-sensitive C27, highly heat-sensitive C30, heat-sensitive C32 and highly heat-sensitive C35) reached a 100.00% YR by the end of the treatment, with their DR varying significantly from 2.00% (C27) to 88.90% (C15). These results demonstrated that although the controlled environment experiment generally validated the conclusions of the field-based classification, discrepancies were observed for certain cultivars such as C15, suggesting that its heat tolerance mechanism might be more susceptible to sustained extreme stress. This is consistent with the previous finding that sustained high temperature stress can accelerate leaf senescence and abscission in ornamental plants (Rivero et al., 2007).

### 3.3 Physiological responses of four *Oncidium* cultivars under graded temperature stress

**Figure 3:**
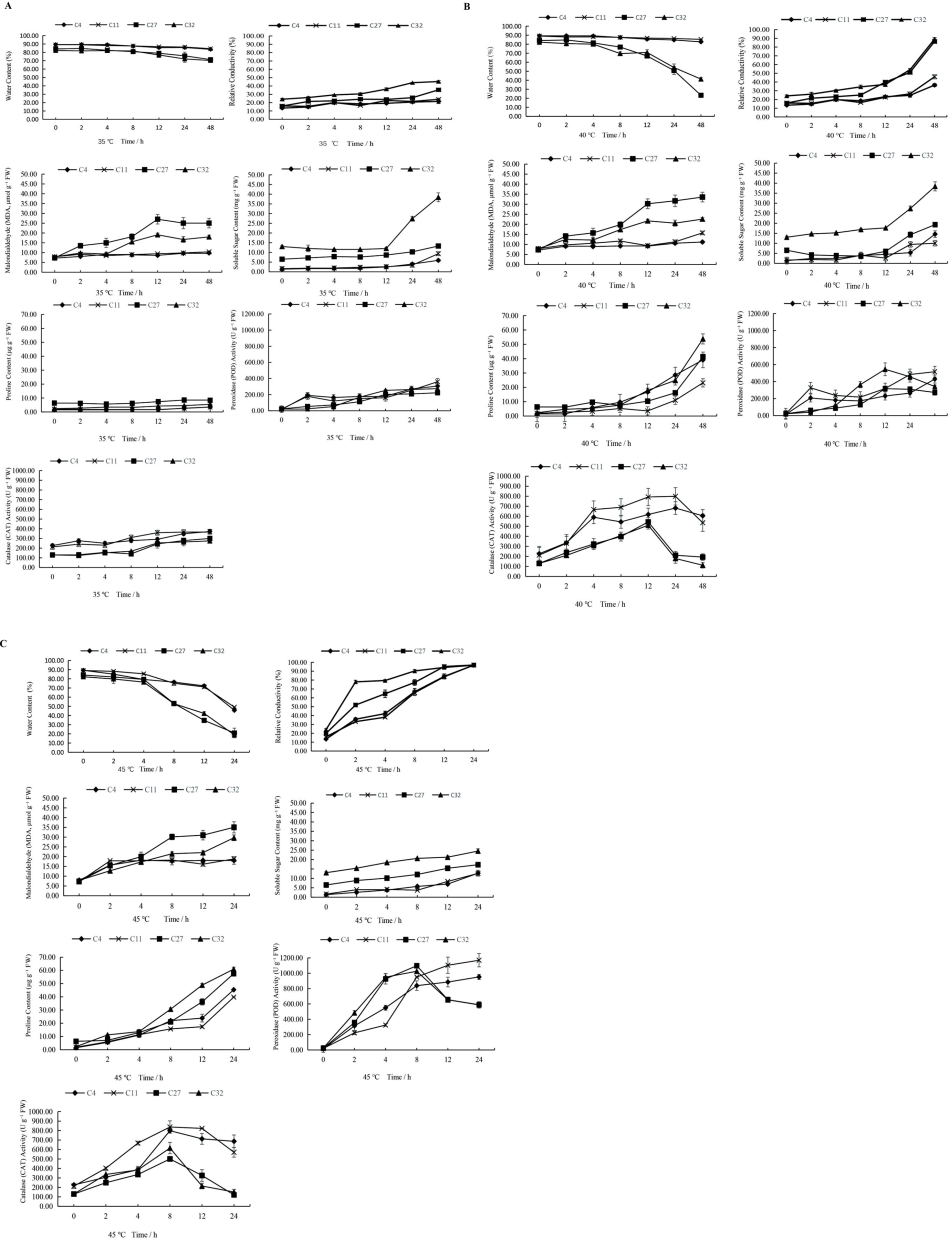
**(A)** Time-course changes of seven physiological indicators in four *Oncidium* cultivars under 35 °C heat stress: leaf water content (LWC, %), relative electrolyte conductivity (REC, %), malondialdehyde (MDA, μmol g^-^¹ FW), proline content (μg g^-^¹ FW), soluble sugar content (mg g^-^¹ FW), peroxidase (POD, U g^-^¹ FW) activity, and catalase (CAT, U g^-^¹ FW) activity. Four cultivars were compared: C4 (heat-tolerant), C11 (highly heat-tolerant), C27 (highly heat-sensitive), and C32 (heat-sensitive). Data were collected at 0, 2, 4, 8, 12, 24, and 48 h after stress initiation. Values are presented as mean ± SD (n = 3 biological replicates). At 35 C, all cultivars maintained relatively high leaf water content (>70%) and low REC (<36%), with only small differences among cultivars, indicating that 35 °C represents mild stress that does not fully reveal genotypic variation in heat tolerance. **(B)** Time-course changes of seven physiological indicators in four *Oncidium* cultivars under 40 C heat stress: leaf water content (LWC, %), relative electrolyte conductivity (REC, %), malondialdehyde (MDA, μmol g^-^¹ FW), proline content (μg g^-^¹ FW), soluble sugar content (mg g^-^¹ FW), peroxidase (POD, U g^-^¹ FW) activity, and catalase (CAT, U g^-^¹ FW) activity. Four cultivars were compared: C4 (heat-tolerant), C11 (highly heat-tolerant), C27 (highly heat-sensitive), and C32 (heat-sensitive). Data were collected at 0, 2, 4, 8, 12, 24, and 48 h after stress initiation. Values are presented as mean ± SD (n = 3 biological replicates). This temperature generated the most pronounced genotypic differences: heat-tolerant cultivars (C4 and C11) maintained significantly higher leaf water content (82–85% at 48 h) and lower REC (31–49%) compared to heat-sensitive cultivars (C27 and C32), which showed severe dehydration (23–41% water content) and membrane damage (88–92% REC). Proline accumulation and POD activity also showed marked differences between tolerant and sensitive cultivars at 40 C. **(C)** Time-course changes of seven physiological indicators in four *Oncidium* cultivars under 45 C heat stress: leaf water content (LWC, %), relative electrolyte conductivity (REC, %), malondialdehyde (MDA, μmol g^-^¹ FW), proline content (μg g^-^¹ FW), soluble sugar content (mg g^-^¹ FW), peroxidase (POD, U g^-^¹ FW) activity, and catalase (CAT, U g^-^¹ FW) activity. Four cultivars were compared: C4 (heat-tolerant), C11 (highly heat-tolerant),C27 (highly heat-sensitive), and C32 (heat-sensitive). Data were collected at 0, 2, 4, 8, 12, and 24 h after stress initiation; sampling at 48 h was omitted due to complete plant desiccation and tissue necrosis. Values are presented as mean ± SD (n = 3 biological replicates). At this extreme temperature, all cultivars underwent rapid physiological decline, with heat-sensitive cultivars reaching near-complete membrane disruption (REC > 96%) and severe water loss (LWC < 25%) within 24 h. Even heat-tolerant cultivars suffered substantial damage, indicating that 45 °C exceeds the critical thermal threshold for *Oncidium* and causes near-universal physiological collapse regardless of genotype.

**Figure 3** presents the time courses of seven physiological indicators in the four cultivars under three temperature treatments, organized into three subplots by temperature for improved readability: 35 °C (**Figure 3A**), 40 °C (**Figure 3B**), and 45 °C (**Figure 3C**). To further elucidate the physiological mechanisms underlying differential heat tolerance, four representative cultivars with distinct heat tolerance gradients were selected from the validation panel: C11 (highly heat-tolerant), C4 (heat-tolerant), C32 (heat-sensitive), and C27 (highly heat-sensitive). These cultivars were subjected to three graded temperature treatments (35.00 °C, moderate stress; 40.00 °C, critical stress; 45.00 °C, extreme stress), with a control group maintained at 22.00 °C, and key physiological indices were measured at multiple time points after stress onset.

#### 3.3.1 Leaf water content under high temperature stress

Heat–tolerant cultivars exhibited significantly stronger leaf water retention capacity than heat–sensitive cultivars under high temperature stress, and the genotypic difference was most pronounced under 40.00 °C critical stress. As shown in **Figure 3**, leaf water content (LWC) declined in all cultivars with increasing temperature and exposure duration, compared with the initial 0 h data of the same treatment. At 35.00 °C (**Figure 3A**), all cultivars maintained relatively high LWC (>70.00%), with heat–tolerant cultivars C4 and C11 showing the greatest stability (83.10–89.60% and 84.40–89.00%, respectively), while heat–sensitive cultivars C27 and C32 exhibited a moderate decline (71.40–84.10% and 70.60–84.20%, respectively). Under 40.00 °C stress (**Figure 3B**), significant moisture loss occurred in sensitive cultivars: the most pronounced reduction was observed in C27 and C32, with LWC decreasing to as low as 23.40% and 41.30% after 48 h, respectively. In contrast, C4 and C11 maintained significantly higher water retention (82.60% and 85.40%, respectively, after 48 h). At 45.00 °C (**Figure 3C**), all cultivars showed rapid dehydration. After 24 h, C27 and C32 exhibited severe water loss, with LWC decreasing to 21.00% and 18.90%, representing reductions of 75.00% and 77.00% compared to their respective controls. In comparison, C4 and C11 retained higher water content (45.70% and 49.10%, respectively) under the same conditions.

#### 3.3.2 Membrane stability under high temperature stress

Membrane stability (measured as relative electrolyte conductivity, REC) was the most intuitive indicator for differentiating heat–tolerant and heat–sensitive cultivars, with heat–tolerant cultivars maintaining significantly lower REC under high temperature stress compared with the initial 0 h data of the same treatment. As shown in **Figure 3**, under 35.00 °C stress (**Figure 3A**), REC of all cultivars remained stable during the first 24 hours, with no significant differences observed between heat–tolerant (C4 and C11) and heat–sensitive (C27 and C32) cultivars. After 48 hours, a moderate increase in REC was observed across all cultivars, with C27 and C32 exhibiting the highest values (35.30–36.00%), suggesting progressive membrane damage under prolonged stress. At 40.00 °C (**Figure 3B**), REC increased sharply in heat–sensitive cultivars: C27 and C32 experienced a dramatic surge to 88.30–92.00% after 48 hours, indicating severe loss of membrane integrity. In contrast, heat–tolerant cultivars C11 and C4 maintained significantly lower REC values (31.30–48.60%), demonstrating superior membrane thermostability. Under 45.00 °C treatment (**Figure 3C**), C27 and C32 reached critical REC levels (96.50–96.90%) within 24 hours, while cultivars C11 andC4 peaked at 96.90–97.00% after 24–48 hours. Notably, the REC of heat–tolerant cultivars increased at a slower rate, underscoring their potential capacity to mitigate heat–induced cellular damage.

#### 3.3.3 Oxidative damage (MDA) under high temperature stress

Heat-tolerant cultivars exhibited significantly weaker oxidative membrane damage than heat-sensitive cultivars under high temperature stress compared with the initial 0 h data of the same treatment, with MDA content consistently lower in tolerant genotypes. Malondialdehyde (MDA) content, a biomarker of lipid peroxidation, exhibited genotype- and temperature-dependent accumulation patterns. At 35.00 °C (**Figure 3A**), heat-tolerant cultivars C4 and C11 showed only a slight increase in MDA content from 0 h to 48 h. In contrast, heat-sensitive cultivars C27 and C32 exhibited a sharp increase, reaching 25.04 ± 0.71 μmol g^-^¹ FW and 18.02 ± 1.27 μmol g^-^¹ FW by 48 h, respectively. Under 40.00 °C stress (**Figure 3B**), the MDA content in C27 and C32 accelerated further, reaching 33.58 ± 0.64 μmol g^-^¹ FW and 22.60 ± 1.75 μmol g^-^¹ FW by 48 h, whereas C11 and C4 maintained significantly lower levels (11.20 ± 1.02 μmol g^-^¹ FW and 15.73 ± 1.23 μmol g^-^¹ FW, respectively). At 45.00 °C stress (**Figure 3C**), C32 and C27 reached critically high MDA levels (29.69 ± 0.64 μmol g^-^¹ FW and 35.04 ± 1.89 μmol g^-^¹ FW) by 24 h, representing a 3.5-fold increase from baseline. The MDA content in C4 and C11 under the same condition was 18.07 ± 3.72 μmol g^-^¹ FW and 18.85 ± 4.25 μmol g^-^¹ FW, respectively.

#### 3.3.4 Osmoprotectant accumulation under high temperature stress

Contrary to the conventional view that “higher proline and soluble sugar accumulation represents stronger heat tolerance”, this study observed that heat-sensitive/highly heat-sensitive cultivars accumulated significantly higher levels of proline and soluble sugars under high temperature stress compared with the initial 0 h data of the same treatment, especially under extreme stress. This phenomenon is likely a passive compensatory response after severe cell damage and dehydration, rather than a proactive osmotic adjustment to enhance heat tolerance.

The accumulation patterns of the osmoprotectants proline and soluble sugars varied significantly among cultivars in response to temperature stress. Under 45.00 °C stress for 24 h, the highest proline content was observed in the heat-sensitive cultivar C32 (60.94 ± 2. 10 μg g^-^¹ FW), followed by the highly heat-sensitive C27 (57.53 ± 0.89 μg g^-^¹ FW). The heat-tolerant cultivars C4 and C11 accumulated substantially less proline (45.37 ± 0.23 μg g^-^¹ FW and 39.77 ± 1.78 μg g^-^¹ FW, respectively). A similar trend was observed for soluble sugars, with C32 and C27 accumulating the highest levels (24.51 ± 1.05 mg g^-^¹ FW and 17.26 ± 0. 10 mg g^-^¹ FW, respectively), compared to C4 and C11 (12.75 ± 1.45 mg g^-^¹ FW and 12.63 ± 1. 12 mg g^-^¹ FW, respectively). The significantly higher accumulation of osmoprotectants in sensitive cultivars was accompanied by severe leaf dehydration, membrane damage and oxidative stress, further suggesting that this may represent a passive stress response after cell injury, rather than an effective protective adaptive mechanism (Krasensky and Jonak, 2012).

#### 3.3.5 Antioxidant enzyme activity in response to high temperature stress

The sustained high activity of antioxidant enzymes (especially peroxidase, POD and catalase, CAT) under high temperature stress was a key characteristic of heat-tolerant *Oncidium* cultivars compared with the initial 0 h data of the same treatment, which was significantly different from the transient increase followed by a sharp decline in sensitive cultivars. As shown in **Figure 3**, the activities of the antioxidant enzymes POD and CAT exhibited distinct responses among the four cultivars under different temperature stresses, with clear differentiation between heat-tolerant (C4, C11) and heat-sensitive (C27, C32) genotypes.

Under 35.00 °C stress (**Figure 3A**), heat-tolerant cultivars C4 and C11 showed sustained increases in both POD and CAT activities over 48 h. In comparison, heat-sensitive cultivars C27 and C32 exhibited more moderate induction. At 40.00 °C (**Figure 3B**), POD activity was strongly induced in all cultivars, with C11 showing the highest sustained activity (516. 13 U g^-^¹ FW at 48 h). CAT activity in heat-tolerant cultivars remained high, with C11 peaking at 799.02 U g^-^¹ FW at 24 h. In contrast, both heat-sensitive cultivars showed declining CAT activity after 12 h. Under the severe 45.00 °C stress (**Figure 3C**), the heat-tolerant cultivars C4 and C11 demonstrated sustained increases in POD activity throughout the 24 h treatment and maintained high CAT activity. Conversely, the heat-sensitive cultivars C27 and C32 showed an early peak in POD activity followed by a significant decline, and suffered a catastrophic collapse in CAT activity, which plummeted to approximately 120.00–150.00 U g^-^¹ FW by 24 h.

### 3.4 Comparative analysis of physiological responses in four *Oncidium* cultivars under high temperature stress

#### 3.4.1 Three-way analysis of variance (ANOVA)

To quantitatively dissect the independent and interactive contributions of genotype, heat stress intensity, and stress duration to physiological variation, a three-way ANOVA was conducted on all measured indices. The results revealed highly significant effects of cultivar, temperature, time, and all their interaction terms on each of the seven physiological traits, including relative electrolyte conductivity (REC), leaf water content (LWC), malondialdehyde (MDA) content, proline content, soluble sugar content, peroxidase (POD) activity, and catalase (CAT) activity.

The key outcomes are summarized as follows:

Cultivar (genotype) effect: A highly significant main effect was detected for all seven traits (P < 0.001), confirming the presence of stable, genetically based differences in heat tolerance among the four *Oncidium* cultivars.

Temperature effect: A highly significant main effect was observed for all indices (P < 0.001), indicating that heat stress intensity acted as the dominant driver of physiological perturbation.

Time effect: A highly significant main effect was found for all parameters (P < 0.001), demonstrating progressive physiological impairment with prolonged heat exposure.

Cultivar × Temperature interaction: Highly significant for all traits (P < 0.001), revealing that cultivars deployed distinct physiological strategies in response to rising temperatures.

Cultivar × Time interaction: Significant for all indices (P < 0.05), indicating that the rate and magnitude of physiological decline differed markedly across cultivars over time.

Temperature × Time interaction: Highly significant for all parameters (P < 0.001), showing that longer stress duration strongly exacerbated the damaging impacts of high temperature.

Cultivar × Temperature × Time interaction: Significant for all physiological traits (P < 0.05), supporting the conclusion that heat tolerance in *Oncidium* is governed by complex, multi-layered regulatory interactions among genotype, environment, and stress duration.

Notably, the highly significant Cultivar × Temperature interaction (P < 0.001) represents the most biologically meaningful result from this analysis, as it formally validates that the observed physiological differences reflect genuine genetic variation rather than stochastic environmental noise. Detailed F-values, degrees of freedom (df), and significance levels (P-values) are provided in **Supplementary Table 4**.

#### 3.4.2 Interrelationships among physiological indicators under high-temperature stress

**Figure 4:**
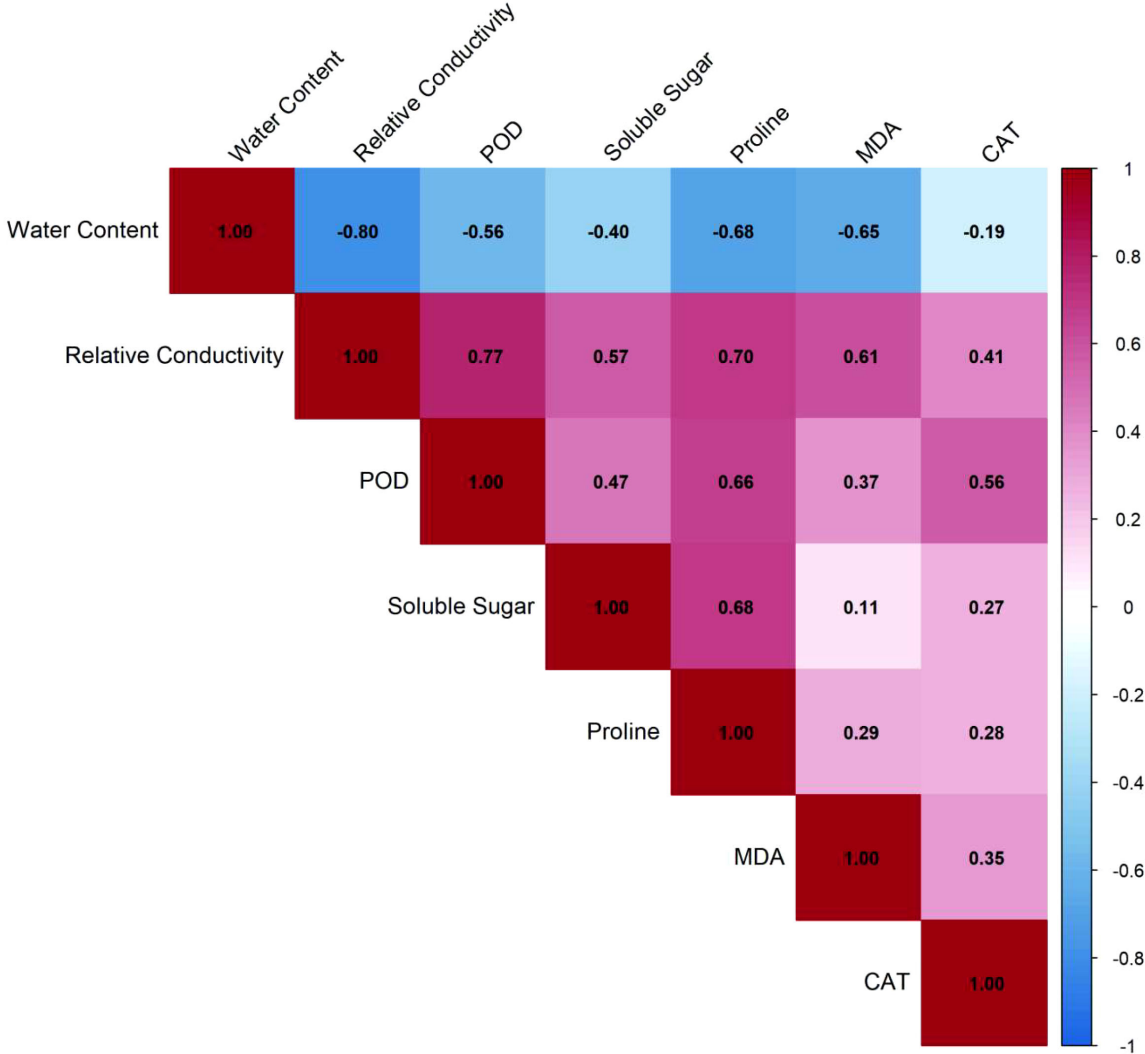
Correlation matrix of physiological indicators in *Oncidium* orchids under high temperature stress. The heat map displays Pearson correlation coefficients among seven physiological indicators measured across four cultivars under three temperature treatments (35.00 C, 40.00 C, and 45.00 C). Red colors indicate positive correlations, blue colors indicate negative correlations, with color intensity proportional to correlation strength.

Pearson correlation analysis revealed that the physiological response to high temperature stress in *Oncidium* formed a tightly coordinated regulatory network, with REC, proline content and POD activity as the core hub indicators. The correlation matrix showed a complex network of significant relationships among the seven measured physiological indicators (**Figure 4**).

Leaf water content (LWC) exhibited the strongest significant negative correlation with REC (r = –0.80, p < 0.001), indicating a tight coupling between cellular dehydration and membrane damage. This relationship was further supported by significant negative correlations between LWC and MDA content (r = –0.65, p < 0.001) and proline content (r = –0.68, p < 0.001), suggesting coordinated responses involving hydration status, oxidative membrane damage, and osmotic adjustment.

REC, as an indicator of membrane permeability, showed significant positive correlations with key stress–responsive indicators, most notably with POD activity (r = 0.77, p < 0.001) and proline content (r = 0.70, p < 0.001). This pattern suggests that the degree of membrane damage is closely associated with the induction of both antioxidant defense and osmotic adjustment pathways.

Among the antioxidant enzymes, POD activity demonstrated the most extensive correlative network, with strong significant positive relationships not only with REC but also with proline content (r = 0.66, p < 0.001). In contrast, CAT activity exhibited a more limited correlation pattern, showing only a moderate significant positive correlation with POD activity (r = 0.56, p < 0.001).

The osmotic adjustment markers proline and soluble sugar content were strongly positively correlated with each other (r = 0.68, p < 0.001), indicating a coordinated accumulation of these osmolytes under high-temperature stress. Two particularly strong correlations (|r| > 0.75) dominated the network: the negative relationship between LWC and REC, and the positive relationship between REC and POD activity. These core interconnections suggested that leaf water status, membrane integrity, and POD activity formed a central response module to high-temperature stress in *Oncidium*.

Overall, the correlation matrix revealed that the physiological response to high-temperature stress involves a tightly coordinated network spanning water relations, membrane stability, oxidative damage, antioxidant defense, and osmotic adjustment. Within this network, relative electrolyte conductivity, POD activity, and proline content appeared to serve as central hubs, linking multiple aspects of the heat stress response.

#### 3.4.3 Principal component analysis

Principal Component Analysis (PCA) further confirmed that membrane stability and osmotic adjustment were the primary determinants of *Oncidium* heat adaptation, with the first two principal components explaining 80.95% of the total variance in physiological indicators. PCA was conducted to discern the underlying patterns in the physiological data, including seven core indicators: Leaf water content, Relative conductivity, MDA, Soluble sugar, Proline, POD, and CAT.

Eigenvalues and variance contributions are summarized in **Table 3**. The first principal component (PC1) accounted for 60.78% of the total variance. The second principal component (PC2) explained an additional 20.17%, resulting in a cumulative variance of 80.95% for the first two dimensions. The first three components together captured 87.36% of the total variance, indicating that they effectively represented the majority of the information contained in the original dataset. The contribution of each variable to the main principal components is detailed in **Table 4**. Variable loadings are presented in **Table 5**. PC1 exhibited strong positive loadings for REC (0.96), proline content (0.89), and MDA content (0.80), but a strong negative loading for leaf water content (–0.91). This pattern defines PC1 as a comprehensive gradient of stress severity, marked by elevated electrolyte leakage, proline and MDA accumulation, and concurrent cellular dehydration. PC2 was distinguished by a very strong positive loading for CAT activity (0.92) and a moderate positive loading for POD activity (0.52), indicating that this axis primarily reflects antioxidant enzyme function. A permutation test (1,000 permutations) confirmed that the mean CV at 40 °C (70.50%) was significantly higher than at 35 °C (57.30%, p = 0.008) and at 45 °C (60.30%, p = 0.021). A weighted discriminability index using PC1 loadings as weights further verified that 40 °C possessed the optimal genotype discriminative capacity (**Supplementary Table 3**).

**Table 3:** Eigenvalues and variance explained by principal components.

Principal component	Eigenvalue	Explained variance (%)	Cumulative variance (%)
PC1	4.25	60.78	60.78
PC2	1.41	20.17	80.95
PC3	0.45	6.41	87.36
PC4	0.36	5.09	92.45
PC5	0.30	4.31	96.76
PC6	0.16	2.24	99.00
PC7	0.07	1.00	100.00
Total	7	100.00	

Principal components (PCs) with eigenvalues > 1.00 (Kaiser criterion) were retained for further analysis. The first two PCs (PC1 and PC2) explained 80.95% of the total variance. The contribution of each variable to the first two principal components is detailed in **Table 4**. For PC1, the variables with the highest contributions were relative conductivity (21.45%), proline (18.55%), and water content (19.51%). For PC2, CAT (59.31%) was the dominant contributor, followed by POD (19. 16%) and soluble sugar (17.29%).

**Table 4:** Contribution (%) of variables to the main principal components.

Variable	Contribution to PC1 (%)	Contribution to PC2 (%)	Contribution to PC3 (%)	Contribution to PC4 (%)	Contribution to PC5 (%)
Relative conductivity	**21.45**	0.31	0.34	1.21	9.32
Proline	18.55	0. 11	0.00	**33.20**	15.08
Water content	19.51	1.87	1.10	8.63	0.46
MDA	15.02	1.94	**47.64**	30.44	2.67
POD	13.63	19.16	1.35	1.42	**40.10**
Soluble sugar	10.81	17.29	47.10	20.77	3.31
CAT	1.03	**59.31**	2.47	4.35	29.06

Variables are ranked in descending order of their contribution to PC1 (the primary dimension of variation). For each principal component (PC), the variable with the highest contribution is highlighted in bold.

**Table 5:** Factor loadings of physiological and biochemical variables on the first five principal components (PC1–PC5).

Variable	PC1	PC2	PC3	PC4	PC5
Relative electrolyte leakage	0.96	0.07	0.04	-0.07	-0.17
Proline content	0.89	-0.04	0	-0.34	0.21
Leaf water content	-0.91	0.16	0.07	0.18	-0.04
Malondialdehyde (MDA) content	0.8	-0.17	-0.46	0.33	0.09
Peroxidase (POD) activity	0.76	0.52	0.08	0.07	-0.35
Soluble sugar content	0.68	-0.49	0.46	0.27	0.1
Catalase (CAT) activity	0.21	0.92	0.11	0.12	0.3

Variables are listed in descending order of the absolute value of their loading on PC1. For each principal component (PC), the variable with the highest absolute loading (defining the axis) is highlighted in bold. Loadings with an absolute value > 0.75 are generally considered strongly associated with the component. MDA, malondialdehyde; POD, peroxidase; CAT, catalase.

#### 3.4.4 Identification of the optimal temperature for high-temperature tolerance evaluation in *Oncidium* orchids

Coefficient of variation (CV) analysis was used to identify the temperature condition that maximally discriminates genotypic differences in heat tolerance among the three tested levels: 35.00 °C, 40.00 °C, and 45.00 °C.

**Figure 5:**
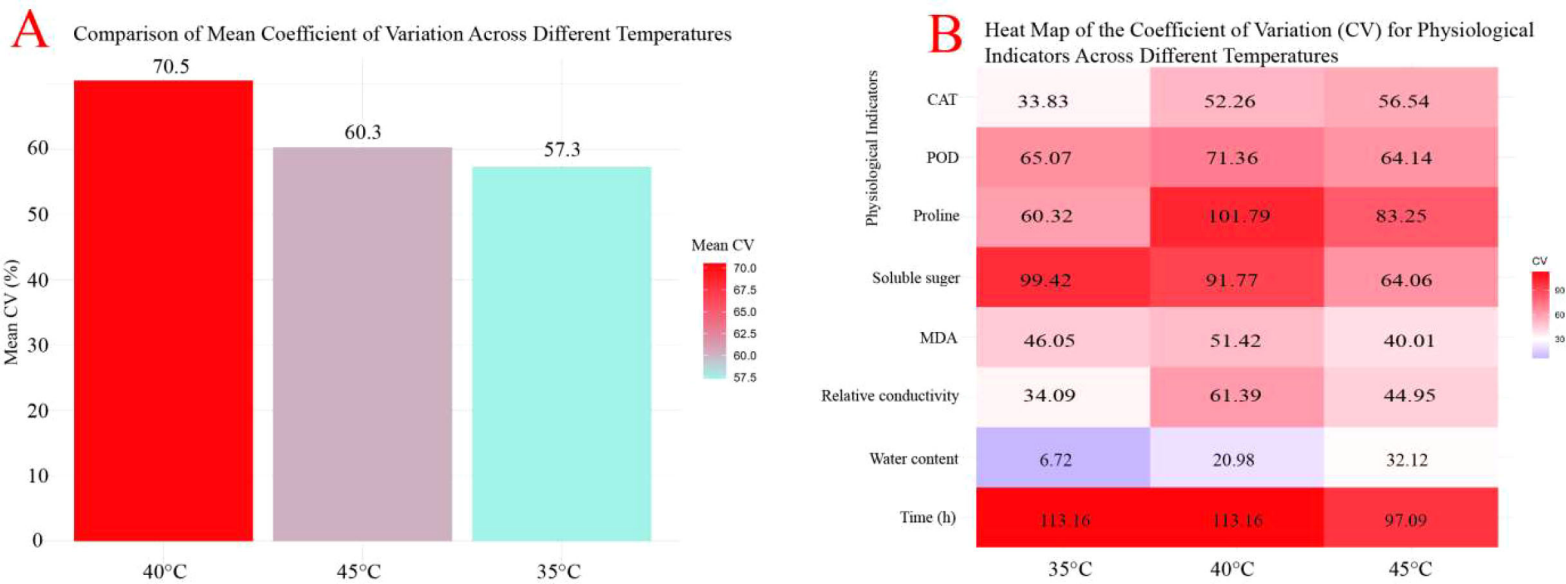
**(A)** Comparison of the mean coefficient of variation (CV, %) across different temperature conditions. **(B)** Heat map of the coefficient of variation (CV) for various physiological indicators under different temperatures and time points. The CV quantifies the disparity among plant varieties under each condition, with a higher CV value (represented by a darker color on the scale) indicating greater inter–cultivar variability.

The 40.00 °C treatment yielded the highest overall discriminatory power across cultivars, with a mean CV of 70.50%—substantially exceeding values at 45.00 °C (60.30%) and 35.00 °C (57.30%) (**Figure 5A**). This result establishes 40.00 °C as the most effective temperature for revealing genetically based differences in physiological heat responses.

At 40.00 °C, five physiological parameters displayed exceptional discriminatory power (CV > 50.00%), making them robust indicators for cultivar classification (**Figure 5B**): proline content (CV = 101.79%), soluble sugar content (91.77%), POD activity (71.36%), REC (61.39%), and CAT activity (52.26%). Among these, REC, proline content, and POD activity emerged as the core diagnostic markers, showing the strongest associations with heat tolerance class and providing practically useful metrics for rapid germplasm screening in *Oncidium* breeding. Collectively, these markers capture critical dimensions of heat tolerance, including osmotic adjustment capacity, antioxidant defense efficiency, and membrane thermostability.

## 4 Discussion

Global warming and frequent extreme heatwaves affect plant growth and productivity. In *Oncidium*, the physiological basis of heat tolerance has not been fully characterized. This study provides a comprehensive evaluation of heat tolerance across 36 *Oncidium* cultivars by combining field screening under natural heatwave conditions with controlled environment validation, followed by in-depth physiological dissection of four representative cultivars with contrasting heat tolerance using multi-trait time-course analyses. We revealed extensive genotypic variation in heat tolerance within the genus, identified 40.00 °C as the optimal discriminative threshold, and defined relative electrolyte conductivity (REC), proline content, and peroxidase (POD) activity as core physiological markers. More importantly, we uncovered a coordinated physiological network underlying *Oncidium* heat adaptation, with membrane stability and osmotic adjustment as primary determinants. These findings contribute to the fundamental understanding of plant stress responses and provide a physiological framework for future genetic and molecular studies in orchids (Wahid et al., 2007).

### 4.1 Evaluation of high temperature tolerance in *Oncidium* cultivars using integrated phenotypic and physiological markers

The extensive genotypic variation in heat tolerance identified in this study confirms the abundant genetic diversity of heat tolerance within the *Oncidium* genus, which provides a core germplasm basis for heat-resilient cultivar breeding (Reynolds and Langridge, 2016). The field heat tolerance classification results of this study are highly consistent with the heat injury grade-based evaluation results in our previous phenotypic screening study (Luo et al., 2024), which fully verifies the reliability and stability of the germplasm heat tolerance phenotype in this study. More importantly, phenotypic differences observed under field heat stress are ultimately governed by differential physiological regulatory capacity; this study further revealed the coordinated physiological regulatory mechanism behind these phenotypic differences through graded temperature stress tests, which makes up for the deficiency that the previous study only focused on phenotypic evaluation without in-depth mechanism dissection. Unlike previous fragmented studies on limited *Oncidium* germplasms, our classification of 36 cultivars into five heat tolerance grades based on field survival rate completes the heat tolerance grading of 36 main cultivated *Oncidium* varieties in tropical China, and supplements the heat tolerance evaluation dataset of this genus. Notably, the eight highly heat-tolerant cultivars identified (field survival rate ≥ 95.00%) possess key traits such as stable membrane integrity and sustained antioxidant capacity, making them candidate materials for subsequent genetic mapping and transcriptomic studies on the genetic basis of heat tolerance.

We acknowledge that seedling age and cultivation substrate varied among the cultivars (**Supplementary Table 1**), which may influence stress tolerance. While these variations may affect absolute survival rates, the subsequent controlled environment validation using uniform plant material (Section 2.2) confirmed the relative heat tolerance ranking for most cultivars. The discrepancy in heat tolerance performance of cultivar C15 between field fluctuating high temperature and controlled constant high temperature provides insight into the mechanistic basis of heat adaptation. C15 showed high survival (83.33%) under field conditions with diurnal temperature fluctuations, but severe leaf yellowing (63.5% YR at week 2) and defoliation (88.9% DR by week 4) under constant 42/38 °C stress. This suggests that C15’s heat tolerance mechanism relies on acclimation and recovery capacity during cooler periods, rather than endurance to sustained extreme heat (Mittler, 2006; White et al., 2012). This observation underscores the importance of multi-environment validation in heat tolerance assessment.

This finding underscores that multi–environment validation is indispensable in *Oncidium* heat tolerance breeding: controlled constant high temperature assay is suitable for rapid elimination of extremely sensitive germplasms in the early breeding stage, while natural field heatwave screening is the gold standard for final validation of varieties suitable for large-scale field promotion. This two-step evaluation strategy is conducive to improving the efficiency and accuracy of *Oncidium* heat tolerance breeding, and can reduce the mismatch between laboratory screening results and field production performance (Gosa et al., 2019).

### 4.2 A coordinated physiological network underpins heat tolerance in *Oncidium*

Plant abiotic stress adaptation relies on synergistic crosstalk among multiple physiological pathways rather than independent individual responses. Superior heat tolerance in *Oncidium* is not determined by a single physiological trait, but by the synergistic operation of a multi-dimensional regulatory network covering water status maintenance, membrane stability, antioxidant defense, and osmotic adjustment, which is consistent with the regulatory pattern of heat adaptation in other *Orchidaceae* species and horticultural crops (Mantri et al., 2011; Demidchik, 2015).

Cell membrane thermostability and water retention represent the frontline physiological defense against heat damage in ornamental plants. Leaf water retention capacity and membrane thermostability constitute the core foundation of *Oncidium* heat adaptation. Coordinated changes in leaf water status and membrane integrity represent universal thermal injury characteristics in perennial horticultural plants. Consistent with previous studies on orchids and carrots, electrolyte leakage acts as a sensitive, early physiological marker for thermal-induced cellular damage across diverse plant species (Nijabat et al., 2020; Fan et al., 2023). The stable low REC in heat-tolerant cultivars is consistent with our previous transcriptomic findings, in which genes related to membrane repair and lipid metabolism were significantly up-regulated in heat-tolerant *Oncidium* cultivars under high temperature stress (Luo et al., 2025), further indicating that membrane thermostability is an important trait for heat adaptation in *Oncidium*. Consistent with previous findings on Phalaenopsis heat tolerance (Blischak, 2005), our results confirm that membrane stability is a core determinant of orchid heat adaptation, while the passive accumulation of proline in sensitive cultivars extends this understanding to *Oncidium*. Unlike most physiological indicators that require complex detection, REC can be measured through a simple, low–cost, and non–lethal sampling procedure, which makes it the most ideal marker for large–scale breeding population screening. This is consistent with the wide application of REC as a core heat tolerance screening marker in cereal crops and legumes; this study further verified its reliability as a heat tolerance evaluation indicator in *Oncidium* (Martineau et al., 1979; Blum and Ebercon, 1981).

Genotype-dependent antioxidant homeostasis is a critical determinant of differential heat resilience under prolonged thermal stress. The genotype–specific response pattern of antioxidant enzymes further reveals the key mechanism of heat tolerance differentiation: heat–tolerant *Oncidium* cultivars can maintain the sustained high activity of POD and CAT under prolonged and extreme high temperature stress, while heat–sensitive cultivars exhibit a transient early induction followed by a catastrophic collapse of enzyme activity. This pattern indicates that the sustained activation of the antioxidant defense system, rather than the short-term transient response, is the key to mitigating heat-induced oxidative damage in *Oncidium* (Suzuki et al., 2012; Hasanuzzaman et al., 2020).

Consistent with our previous transcriptomic study (Luo et al., 2025), the sustained high activity of POD and CAT in heat-tolerant cultivars corresponds to the significant up-regulation of their encoding genes under high temperature stress, indicating that the sustained activation of antioxidant enzyme genes is the molecular basis for the stable antioxidant capacity of heat-tolerant cultivars. The variable antioxidant response magnitude across genotypes further validates the auxiliary reference value of POD for germplasm heat tolerance evaluation.

Osmolyte accumulation under heat stress exhibits dual functional patterns, including active protective regulation and passive damage-associated accumulation. A notable observation of this study is that heat-sensitive cultivars accumulated significantly higher levels of proline and soluble sugars under extreme high temperature stress, which appears inconsistent with the traditional view that higher osmolyte accumulation represents stronger stress tolerance. This phenomenon is not unique to *Oncidium*: similar results have been reported in other *Orchidaceae* species such as *Dendrobium nobile*, where sensitive cultivars showed excessive proline accumulation under severe high temperature stress (Fan et al., 2023), as well as in horticultural crops such as chrysanthemum and rose, where high osmolyte levels were found to be a symptom of severe cell damage rather than a protective adaptation (Rivero et al., 2007; Krasensky and Jonak, 2012).

This dual regulatory mode of osmotic adjustment under thermal stress has been systematically summarized in classic plant stress physiology reviews (Krasensky and Jonak, 2012). Our results support a distinction between active protective accumulation and passive damage-induced accumulation of osmolytes: the former occurs in the early stage of mild to moderate stress, is accompanied by stable membrane integrity and normal cell metabolism, and functions to maintain cellular osmotic balance (Blum, 2017); the latter tends to occurs in the late stage of extreme stress, is significantly positively correlated with REC and MDA content (indicators of cell damage), and may reflect essentially a passive compensatory response after catastrophic cell dehydration and metabolic disorder (Szabados and Savouré, 2010). Such phenotypic associations fully support this interpretation, which also explains why proline content, despite its high discriminative power, cannot be used alone as a positive indicator of heat tolerance, and must be evaluated in combination with membrane integrity indicators such as REC.

However, we acknowledge that correlation does not establish causality. While our data are consistent with the interpretation that proline accumulation in sensitive cultivars is a passive response to cell damage, direct evidence—such as measuring proline biosynthesis enzyme activity (P5CS) or expression of proline-related genes—would be required to confirm this mechanism. Future studies will address this limitation.

### 4.3 40.00 °C is the optimal threshold for *Oncidium* heat tolerance phenotyping and cultivation management

Standardized stress temperature conditions are essential to eliminate environmental noise and achieve accurate genotypic physiological differentiation during germplasm phenotyping. The identification of 40.00 °C as the optimal discriminative temperature for *Oncidium* heat tolerance evaluation is a key result of this study, which provides a unified temperature condition reference for the horizontal comparison of heat tolerance among different *Oncidium* germplasms, and supplements the deficiency of inconsistent stress conditions in previous related studies (Araus et al., 2008; Langridge and Reynolds, 2021).

From a mechanistic perspective, this critical temperature threshold enables efficient dissection of heat tolerance physiology: a 48h 40.00 °C treatment combined with measurement of the three core markers (REC, proline, POD) provides a rapid and reference method to quantify genotypic differences in heat adaptation. Compared to traditional long term phenotyping, this approach facilitates efficient screening of large germplasm collections for physiological characterization and subsequent genetic studies.

### 4.4 Limitations of the study and future research directions

This study systematically elucidated the physiological mechanism of heat tolerance in *Oncidium* and established a practical evaluation system. The findings ofthis study provide a theoretical basis and technical reference for heat-tolerant germplasm evaluation, marker-assisted breeding, and precision cultivation management of *Oncidium*. Despite the systematic findings above, there are still some limitations of this study that need to be clarified.

First, this study focused on the heat tolerance of *Oncidium* at the vegetative seedling stage. The flowering stage—the most critical stage for commercial trait formation—is typically far more sensitive to high temperature stress in orchids. For example, in *Phalaenopsis* (the most closely related commercially cultivated orchid genus), temperatures as low as 30–32 °C can cause flower bud abortion, reduced flower size, and shortened vase life, while temperatures above 35 °C lead to complete inflorescence necrosis (Chen et al., 2017; Li et al., 2020). The heat tolerance performance at the seedling stage is not completely consistent with that at the flowering stage. Therefore, the heat tolerance ranking established in this study should be interpreted primarily for the seedling stage, and future studies should evaluate flowering-stage heat tolerance separately.

Second, this study focused exclusively on physiological responses occurring during continuous high-temperature stress, without characterizing post-stress recovery dynamics. The 48–72 h reversibility of critical physiological indices, including REC, MDA, and antioxidant enzyme activities, largely determines plant resilience and regrowth potential after heatwave events, which is essential for commercial orchid production. Therefore, systematic evaluation of post-stress recovery capacity in representative cultivars will be conducted in our future work to complement the present findings.

Third, the physiological assays in this study were conducted under constant high temperature conditions, while the natural field environment is characterized by fluctuating temperatures and combined stresses (high temperature + high light + drought), which may lead to differences in the performance of physiological markers under complex field conditions.

Fourth, this study only revealed the physiological regulatory mechanism of *Oncidium* heat tolerance; while the molecular genetic basis underlying the differential heat resilience among cultivars has been initially explored in our recent transcriptomic study (Luo et al., 2025), the key functional genes and their regulatory networks still need to be further validated.

Fifth, the biomarker panel (REC, proline, and POD) was identified and internally validated using the same four cultivars, which constitutes circular validation. Therefore, prospective testing on an independent set of 5–10 cultivars with known field heat tolerance grades is necessary before these biomarkers can be reliably applied to routine *Oncidium* breeding practice.

To further advance the understanding of heat tolerance mechanisms in *Oncidium*, subsequent research will focus on the following directions. Notably, our team has completed transcriptomic analysis of heat shock responses in *Oncidium* cultivars with significantly differential heat tolerance, and the relevant research results have been formally published (Luo et al., 2025). Currently, we are focusing on mining key candidate genes associated with *Oncidium* heat tolerance based on the published physiological and transcriptomic dataset. The specific research directions include: (1) Establish a flowering-stage heat tolerance evaluation system for *Oncidium* to understand stage-specific responses; (2) Conduct multi-year, multi-site field validation of the core physiological markers to assess their stability under complex environmental conditions; (3) Perform joint transcriptomic and metabolomic analysis of heat-tolerant and sensitive cultivars under critical high temperature stress, to mine key regulatory genes and metabolic pathways underlying heat tolerance in *Oncidium*; (4) Develop Kompetitive Allele-Specific PCR (KASP) molecular markers based on the key physiological markers and candidate genes to facilitate genetic studies; (5) Conduct combining ability analysis of the core heat-tolerant candidate materials identified in this study to construct specialized populations for genetic dissection of heat tolerance (Varshney et al., 2021).

## 5 Conclusion

This study systematically evaluated the heat tolerance of 36 *Oncidium* cultivars through field screening and controlled physiological assays. We identified 40.00 °C as the optimal discriminative threshold for heat tolerance phenotyping, and defined relative electrolyte conductivity (REC), proline content, and peroxidase (POD) activity as core physiological biomarkers. Membrane stability and osmotic adjustment were confirmed as the primary determinants of *Oncidium* heat adaptation. These findings identify physiological traits associated with heat tolerance in *Oncidium* and provide a reference for future studies on stress tolerance in orchids.

## Data Availability

The original contributions presented in the study are included in the article/**Supplementary Material**. Further inquiries can be directed to the corresponding author.

## Supplementary material

Supplementary Table 1 Detailed information of 36 *Oncidium* cultivars used in the field heat tolerance evaluation, including germplasm source, seedling age, cultivation substrate, sample size, field survival rate, heat tolerance classification, and bootstrapped 95% confidence intervals for survival rates.

Supplementary Table 2 Sensitivity analysis of field heat tolerance classification (robustness test of the original classification by excluding cultivars with sample size <10).

Supplementary Table 3 Comparison of equal-weighted mean coefficient of variation (CV) and PCA loadings-based weighted discriminability index across three temperature treatments.

Supplementary Table 4 Three-way ANOVA results for the effects of cultivar, temperature, and treatment time on physiological indicators of *Oncidium* orchids under high-temperature stress.

Supplementary Table 5 Quantitative justification for the selection of four representative cultivars.
