# Supplementary material for: Cultivar differences in heat tolerance of Oncidium orchids: physiological mechanisms and implications for breeding strategies
Source: Front Plant Sci. 2026 May 22;17:1831843. doi: 10.3389/fpls.2026.1831843 (PMC13236530; doi:10.3389/fpls.2026.1831843)
Supplement: Supplementary Table 1 — Detailed information of 36 Oncidium cultivars used in the field heat tolerance evaluation, including germplasm source, seedling age, cultivation substrate, sample size, field survival rate, heat tolerance classification, and bootstrapped 95% confidence intervals for survival rates. [file DataSheet1.docx]

Table S1 Detailed information of 36 Oncidium cultivars used in the field heat tolerance evaluation, including germplasm source, seedling age, cultivation substrate, sample size, field survival rate, heat tolerance classification, and bootstrapped 95% confidence intervals for survival rates.

| **Variety No.** | **Variety Name** | **Germplasm Source** | **Seedling Age** | **Cultivation Substrate** | **Bootstrapped 95% CI (%)** |
| --- | --- | --- | --- | --- | --- |
| C1 | Oncidium Gower Ramsey‘White Jade’ | Taiwan | 1.5 years | Sphagnum moss | 100.00–100.00 |
| C2 | Oncidium Sweet Sugar‘Angel’ | Guangdong Province | 1 year | Sphagnum moss | 100.00–100.00 |
| C3 | Gomcidumnia Kamphaengsan white | Yunnan Province | 2 years | Sphagnum moss | 100.00–100.00 |
| C4 | Oncidium Sweet Sugar | Haikou | 1.5 years | Sphagnum moss | 86.96–96.52 |
| C5 | Oncidium Gower Ramsey ‘No.3’ | Haikou | 1 year | Sphagnum moss | 100.00–100.00 |
| C6 | Oncidium Tolumnia Snow Dancer | Guangdong Province | 1 year | Sphagnum moss | 100.00–100.00 |
| C7 | Oncidium Sweet Sugar‘Emperor’ | Haikou | 1.5 years | Sphagnum moss | 100.00–100.00 |
| C8 | Oncidium Red M | Yunnan Province | 1.5 years | Sphagnum moss | 92.06–99.74 |
| C9 | Oncidium Gower Ramsey‘Volcano Queen’ | Fujian Province | 2 years | Sphagnum moss | 81.48–98.77 |
| C10 | Oncidesa Gower Ramsey‘Sunkist’ | Fujian Province | 1.5 years | Sphagnum moss | 81.82–97.73 |
| C11 | Oncidium Gower Ramsey 'Lemon Heart' | Haikou | 2 years | Sphagnum moss | 100.00–100.00 |
| C12 | Oncidium Boso Sweet | Haikou | 2 years | Sphagnum moss | 64.10–98.90 |
| C13 | Oncidium Flexuosa | Haikou | 1.5 years | Sphagnum moss | 73.53–93.14 |
| C14 | Burrageara Kilauea‘Hawaii’ | Guangdong Province | 1 year | Sphagnum moss | 62.50–93.75 |
| C15 | Oncidium Aloha Iwanaga | Fujian Province | 1.5 years | Sphagnum moss | 71.43–91.67 |
| C16 | Oncidium Sharry Baby‘Sweet Fragrance’ | Hainan Province | 2 years | Sphagnum moss | 71.43–85.71 |
| C17 | Oncidium Oncsa. Goldiana‘Golden Shower’ | Guangdong Province | 1.5 years | Sphagnum moss | 62.50–91.67 |
| C18 | Oncidium Roseoides | Yunnan Province | 2 years | Sphagnum moss | 50.00–92.86 |
| C19 | Oncidium Zelenkocidium Kukoo | Guizhou Province | 1.5 years | Sphagnum moss | 62.82–79.22 |
| C20 | Oncidium Aka Baby | Hainan Province | 1 year | Sphagnum moss | 61.31–75.00 |
| C21 | Brassia Verrucos | Guangdong Province | 2.5 years | Pine bark | 35.71–78.57 |
| C22 | Oncidium Jairak Frangrance | Haikou | 1 year | Pine bark | 29.17–58.33 |
| C23 | Oncidesa Sotoanum-barbata | Fujian Province | 2 years | Pine bark | 29.17–62.50 |
| C24 | Oncidium Jairak Fragrance (red) | Haikou | 1 year | Sphagnum moss | 35.29–52.94 |
| C25 | Oncidium Sharry Baby | Guangdong Province | 2 years | Pine bark | 25.00–62.50 |
| C26 | Dgmra Winter Wonderland‘White Fairy’ | Fujian Province | 1 year | Sphagnum moss | 27.03–56.76 |
| C27 | Oncidium 'Katrin Zoch' | Fujian Province | 1 year | Sphagnum moss | 0.00–57.14 |
| C28 | Oncidium Jairak Fragrance (pink) | Haikou | 2 years | Pine bark | 26.79–51.79 |
| C29 | Oncidium Little Cherry | Guizhou Province | 1.5 years | Pine bark | 20.41–44.90 |
| C30 | Oncidium Sharry Baby‘Tricolor’ | Fujian Province | 2 years | Pine bark | 11.11–44.44 |
| C31 | Oncidium Twinkle“yellow Fantasy” | Fujian Province | 1 year | Sphagnum moss | 9.09–40.91 |
| C32 | Oncidium Aliceara Tahitian Dancer | Hainan Province | 1.5 years | Pine bark | 25.00–58.33 |
| C33 | Miltonidium Issaku Nagata ‘Volcano Queen’ | Guangdong Province | 1 year | Sphagnum moss | 0.00–0.00 |
| C34 | Oncidesa Hwuluduen Chameleon | Yunnan Province | 1.5 years | Sphagnum moss | 0.00–0.00 |
| C35 | Oncidium Flexuosa yellow | Fujian Province | 1.5 years | Sphagnum moss | 0.00–0.00 |
| C36 | Oncidium Twinkle (Fragrance Fantasy) | Hainan Province | 1 year | Sphagnum moss | 0.00–0.00 |

****Note:**** Bootstrapped 95% confidence intervals (CI) for field survival rates were calculated using the Wilson method via 1000 bootstrap resampling replicates with replacement. All cultivars were classified into five heat tolerance classes: highly tolerant (survival rate ≥ 95.00%), tolerant (80.00–94.00%), moderately tolerant (60.00–79.00%), sensitive (30.00–59.00%), and highly sensitive (survival rate < 30.00%). Some cultivars have small sample sizes due to the limitation of the existing germplasm resource reserve; their heat tolerance classification will be further verified by expanding the sample size in the future.

Note: All cultivars were preserved in the Tropical Flower Research Center, CATAS, Danzhou, Hainan Province.
