# Supplementary material for: Cultivar differences in heat tolerance of Oncidium orchids: physiological mechanisms and implications for breeding strategies
Source: Front Plant Sci. 2026 May 22;17:1831843. doi: 10.3389/fpls.2026.1831843 (PMC13236530; doi:10.3389/fpls.2026.1831843)
Supplement: Supplementary file 2 [file DataSheet2.docx]

### Table S 2 Sensitivity analysis of field heat tolerance classification (robustness test of the original classification by excluding cultivars with sample size < 10).

| **Original heat tolerance class** | **Number of cultivars** | **Number of cultivars with n ≥ 10** | **Number of cultivars with consistent classification** | **Consistency rate** |
| --- | --- | --- | --- | --- |
| Highly tolerant (HT) | 8 | 7 | 7 | 100% |
| Tolerant (T) | 12 | 10 | 10 | 100% |
| Sensitive (S) | 10 | 8 | 8 | 100% |
| Highly sensitive (HS) | 6 | 5 | 5 | 100% |
| **Total** | **36** | **30** | **30** | **100%** |

Note: The original classification was based on survival rate after the 2021 natural heatwave. Sensitivity analysis was performed by excluding six cultivars with sample size < 10 (C7, C14, C19, C28, C33, C35). All remaining 30 cultivars showed identical heat tolerance classification as the original result, confirming the robustness of our field screening.
