# Supplementary material for: Cultivar differences in heat tolerance of Oncidium orchids: physiological mechanisms and implications for breeding strategies
Source: Front Plant Sci. 2026 May 22;17:1831843. doi: 10.3389/fpls.2026.1831843 (PMC13236530; doi:10.3389/fpls.2026.1831843)
Supplement: Supplementary file 3 [file DataSheet3.docx]

Supplementary Table S3. Comparison of equal-weighted mean coefficient of variation (CV) and PCA loadings-based weighted discriminability index across three temperature treatments

| **Treatment temperature** | **Equal-weighted mean CV (%)** | **Weighted discriminability index (%)** | **Permutation test p-value (vs. 40 °C)** |
| --- | --- | --- | --- |
| 35 °C | 57.3 | 56.12 | 0.008 |
| 40 °C | 70.5 | 71.35 | – |
| 45 °C | 60.3 | 59.87 | 0.021 |

Note: The weighted discriminability index was calculated using PC1 loading values as independent weights for seven physiological indices (LWC, REC, MDA, proline, soluble sugar, POD, CAT). PC1 explained 60.78% of the total physiological variation. A total of 1000 random permutation replicates were performed to test the significant differences in mean CV among temperature treatments. Lower p-values indicate significant differences in genotype discriminative capacity relative to the 40 °C treatment.
