# Supplementary material for: Cultivar differences in heat tolerance of Oncidium orchids: physiological mechanisms and implications for breeding strategies
Source: Front Plant Sci. 2026 May 22;17:1831843. doi: 10.3389/fpls.2026.1831843 (PMC13236530; doi:10.3389/fpls.2026.1831843)
Supplement: Supplementary file 4 [file DataSheet4.docx]

### Supplementary Table S4

Three-way ANOVA results for the effects of cultivar, temperature, and treatment time on physiological indicators of Oncidium orchids under high-temperature stress

| **Source of Variation** | **df** | **REC** | **LWC** | **MDA** | **Proline** | **Soluble sugar** | **POD** | **CAT** |
| --- | --- | --- | --- | --- | --- | --- | --- | --- |
| Leaf age (covariate) | 1 | 2.31 ns | 6.78* | 1.92 ns | 2.15 ns | 5.43* | 1.68 ns | 2.03 ns |
| Cultivar (C) | 3 | 116.31*** | 89.22*** | 65.43*** | 140.56*** | 78.94*** | 103.21*** | 89.67*** |
| Temperature (T) | 2 | 198.76*** | 172.35*** | 132.18*** | 227.83*** | 156.72*** | 184.53*** | 162.88*** |
| Time (t) | 5 | 77.92*** | 66.54*** | 55.28*** | 83.96*** | 61.85*** | 71.46*** | 60.23*** |
| C × T | 6 | 69.85*** | 52.18*** | 44.56*** | 80.92*** | 47.35*** | 60.89*** | 49.27*** |
| C × t | 15 | 11.28* | 8.96* | 7.82* | 13.41* | 7.23* | 10.35* | 8.11* |
| T × t | 10 | 62.45*** | 49.87*** | 42.53*** | 73.18*** | 46.92*** | 57.12*** | 51.08*** |
| C × T × t | 30 | 3.89* | 3.45* | 2.92* | 4.78* | 2.71* | 3.72* | 3.25* |
| Error | 503 | – | – | – | – | – | – | – |
| Total | 575 | – | – | – | – | – | – | – |

**Note**: *P < 0.05, ***P < 0.001, ns = not significant. REC: relative electrolyte conductivity; LWC: leaf water content; MDA: malondialdehyde; POD: peroxidase; CAT: catalase. Leaf age (node position) was included as a covariate to control for developmental heterogeneity in physiological responses. Although leaf age had a small significant effect on LWC and soluble sugar content, all original main effects and the key cultivar × temperature interaction remained highly significant and qualitatively unchanged.
