# Supplementary material for: Cultivar differences in heat tolerance of Oncidium orchids: physiological mechanisms and implications for breeding strategies
Source: Front Plant Sci. 2026 May 22;17:1831843. doi: 10.3389/fpls.2026.1831843 (PMC13236530; doi:10.3389/fpls.2026.1831843)
Supplement: Supplementary file 5 [file DataSheet5.docx]

**Supplementary Table S5. Quantitative justification for the selection of four representative cultivars**

| **Heat tolerance class** | **Full survival rate range of 36 cultivars** | **Selected cultivar** | **Field survival rate (%)** | **28-day YR (%)** | **28-day DR (%)** |
| --- | --- | --- | --- | --- | --- |
| Highly heat-tolerant | 95.00–100.00 | C11 | 100 | 2.4 | 0 |
| Heat-tolerant | 80.00–94.00 | C4 | 93.04 | 74.5 | 0 |
| Heat-sensitive | 30.00–59.00 | C32 | 41.67 | 100 | 21.4 |
| Highly heat-sensitive | 0.00–29.99 | C27 | 14.28 | 100 | 2 |

**Note:** YR = leaf yellowing rate; DR = defoliation rate. Data for YR and DR were obtained from the 28-day continuous high-temperature stress experiment (42.00 °C/38.00 °C day/night) presented in Table 2.
